# Brominated Dioxins in Egg, Broiler, and Feed Additives: Significance of Bioassay-Directed Screening for Identification of Emerging Risks in Food

**DOI:** 10.3390/foods13060931

**Published:** 2024-03-19

**Authors:** Caroline Dirks, Arjen Gerssen, Yoran Weide, Thijs Meijer, Guido van der Weg, Milou G. M. van de Schans, Toine F. H. Bovee

**Affiliations:** Wageningen Food Safety Research, 6708 WB Wageningen, The Netherlands; arjen.gerssen@wur.nl (A.G.); yoran.weide@wur.nl (Y.W.); thijs.meijer@wur.nl (T.M.); milou.vandeschans@wur.nl (M.G.M.v.d.S.); toine.bovee@wur.nl (T.F.H.B.)

**Keywords:** bioassay, brominated dioxins, L-lysine, DR CALUX, GC-HRMS, tetrabromo-dibenzofuran, egg, broiler, seaweed, emerging risk

## Abstract

Food authorities aim to safeguard our food. This requires sensitive analyses to guarantee detection of both banned and regulated substances at low concentrations. At the same time, broad screening methods are needed to identify new emerging risks. For this purpose, effect-based bioassays combined with mass spectrometric analyses offer an advantage. During the regular monitoring of dioxins in agricultural products, a discrepancy was observed between the results of the DR CALUX (Dioxin-Responsive Chemical Activated Luciferase gene Expression) bioassay and the confirmatory gas chromatographic high resolution mass spectrometric (GC-HRMS) analysis in egg and broiler fat samples. The response in the bioassay was high, suggesting a clear exceedance of the maximum limits of dioxins in these samples, yet regulated dioxins or dl-PCBs were not detected by GC/HRMS analysis. Ultimately, a broad screening analysis using GC-HRMS resulted in the identification of 2,3,7,8-tetrabromo-dibenzofuran (2,3,7,8-TBDF) in both egg and broiler fat. To investigate the potential source of this brominated furan contaminant, different samples were analyzed: bedding material, poultry feed, feed additives (choline chloride and l-lysine), and seaweed. The poultry feed and feed additives all contained 2,3,7,8-TBDF. Using a feed-to-food transfer model, it became clear that the poultry feed was probably the source of 2,3,7,8-TBDF in broilers and eggs through a feed additive like L-lysine or choline chloride. This study underlines the importance of using a combination of effect-based screening assays with sensitive analytical methods to detect potential new and emerging risks.

## 1. Introduction

In order to guarantee food safety, it is important to monitor the presence of harmful substances throughout the food production chain. With this strategy, various compounds have been identified in food and feed in the past years that could represent a potential threat to human health [1,2,3,4]. One group of compounds that represents continued health concern are the chlorinated dioxins and dioxin-like polychlorinated biphenyls (dl-PCBs). For this reason, these compounds are regulated in feed and food, in Europe since 2011 (EC/277/2012 and EU/2023/915). Dioxins is a collective name for polychlorinated dibenzo-p-dioxins (PCDDs) and polychlorinated dibenzofurans (PCDFs). Dioxins can be formed as by-products from natural and industrial combustion processes and do not have an industrial application [5,6]. They are very persistent and lipophilic and, as a result, are present in the environment worldwide, i.e., from fish and animals to soil and ball clay.

While dioxins and dioxin-like PCBs consist of many congeners, i.e., 75 PCDDs, 135 PCDFs, and 209 PCBs, only those that have chlorines in the lateral positions (2,3,7,8 for PCDDs/Fs and 3,4 and/or 5 for PCBs) are considered toxic. For these toxic congeners the World Health Organization (WHO) provided toxic equivalency factors (TEF) [7]. TEF values were established for 7 PCDDs, 10 PCDFs, and 12 dioxin-like PCBs (dl-PCBs); relative to the most potent dioxin, 2,3,7,8-tetrachlorodibenzo-p-dioxin (TCDD). TCDD is assigned with a TEF value of 1. The toxicity-weighted concentration of mixtures of PCDDs, PCDFs, and PCBs is expressed as TEQ (toxic equivalents). In order to calculate the TEQ value, the concentration of each congener should be multiplied by their TEF values and summed. The equivalent of the TEQ value in Gas Chromatography coupled to high resolution mass spectrometry (GC-HRMS) data is the BEQ (bioanalytical equivalents) value in DR CALUX (Dioxin-Responsive Chemical Activated Luciferase gene Expression). Usually, the TEQ and BEQ values of a sample lie close together because the TEF values, established by the WHO, are similar to the responses in the DR CALUX and result in an added response in the DR CALUX [8]. In case a sample has a BEQ value in the DR CALUX that is substantially higher than the measured TEQ value from the GC-HRMS data, this indicates that another persistent organic pollutant is present in the sample, with the same toxic mode of action as the PCDD/Fs and dl-PCBs.

In European legislation, both action levels as well as maximum levels were set for the total sum (ng TEQ/g) of dioxins and dl-PCBs for food and feed items. The purpose of action levels, which are lower than the maximum levels, is to possibly identify the source of contamination in order to extract this source from the food chain to actively direct toward lower levels of dioxins and dl-PCBs in primary food and feed products and thereby lowering human exposure (EU/2023/915). In order to fulfill the legislation, i.e., guarantee products with safe levels of regulated harmful compounds, and to check on the possible presence of new emerging harmful compounds (potential risks), samples were firstly screened with a cell-based bioassay. Toxicity from dioxins and dioxin-like compounds is mainly initiated via the interaction with the Aryl hydrocarbon Receptor (AhR) in cells [9]. The DR CALUX bioassay used as initial screening in our laboratory is such an AhR bioassay. The outcomes of this screening assay are relevant and worldwide fully accepted by regulatory institutions. Samples eliciting a bioassay response indicating levels above the action limit (suspect) for dioxins and PCBs, were confirmed by GC-HRMS analysis. The confirmation analyses by GC-HRMS have a targeted scope focused on the congeners with established TEF values. Hence, the GC-HRMS targets 17 PCDDs/Fs and 12 dioxin-like PCBs, in accordance with Regulation (EU) 2017/644 for food and Regulation (EC) No 152/2009 for feed. Other chlorinated congeners are routinely not taken into account due to low or absence of TEFs. Also, other halogenated congeners, e.g., brominated, mixed chlorinated/brominated or iodized, are routinely also not taken into account when performing GC-HRMS confirmation analysis. Thus, the most potent 17 PCDDs/Fs and 12 dioxin-like PCBs have been highly regulated and monitored over the years [10,11,12], while their analogue compounds received far less attention and are overseen. These compounds are consequently neither regulated nor monitored [13]. Although an attempt has been made to establish TEF values for the brominated dioxins and dl-PBBs, an expert panel from the World Health Organization (WHO) and United Nations Environment Programme (UNEP) concluded that due to the lack of mammalian in vitro and in vivo data, the TEFs of the chlorinated analogues should be used for human risk assessment for these brominated compounds [14]. Relative potency (REP) values have been determined regarding the AhR activation by experiments with both the rat cell-based (DR CALUX) and the human cell-based (DRhuman CALUX) bioassay [15], and indicated similar TEF values for these brominated compounds as their chlorinated counterparts.. Although not regulated, the brominated dioxins have similar toxicological effects as their chlorinated analogues [16,17].

**Figure 1:**
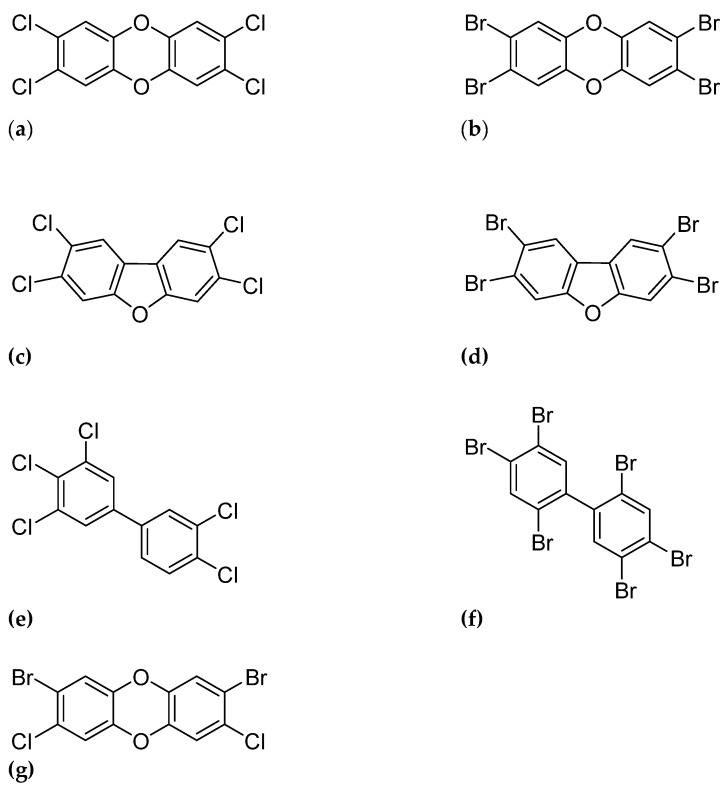
Molecular structures of representatives of the groups PCDD/Fs, PBDD/Fs, dioxin-like PCB’s and mixed PXDD/Fs: (**a**) 2,3,7,8-tetrachloro-dibenzo-p-dioxin (**b**) 2,3,7,8-tetrabromo-dibenzo-p-dioxin (**c**) 2,3,7,8,-tetrachloro-dibenzo-furan (**d**) 2,3,7,8,-tetrabromo-dibenzo-furan (**e**) 3,3′,5,5′-pentachlorobiphenyl (PCB-126) (**f**) 3,3′,5,5′-pentabromobiphenyl (PBB-126) (**g**) 2,8-dibromo-3,7-dichlorodibenzo-p-dioxin.

Similar to the chlorinated dioxins, brominated dioxins can be distinguished into two groups: the polybrominated dibenzo-p-dioxins (PBDDs) and polybrominated dibenzofurans (PBDFs) (Figure 1). Brominated dioxins are by-products from the brominated flame retardant industry—for instance, from the production of polybrominated diphenyl ether (PBDEs) [18]. Mixtures of PBDEs contain significant amounts of PBDD/Fs, especially PBDFs [19]. Moreover, during recycling processes, new PBDD/Fs can be formed [20,21]. There is only a limited number of studies describing the occurrence of brominated dioxins in food and feed [22,23,24,25,26]. Even so, brominated dioxins are found in food commodities such as fish, shellfish, river fish, marine deep sea, salmon, cod liver, meat, animal fat (bovine, ovine, porcine), eggs and poultry, milk and dairy products, fresh vegetables, fruits and nuts, cooking oils, and even bread [27]. The WHO estimated that the dietary intake of PCDD/Fs accounts for approximately 90% of the total human exposure to PCDD/Fs and PBDD/Fs [28].

In poultry egg, the main PBDD/Fs found were 2,3,7,8-TBDF, 1,2,3/4,7,8-PeBDF, 1,2,3,4,7,8-HxBDF and 1,2,3,4,6,7,8-HpBDF and, to a lesser extent, 1,2,3,(4/6),7,8-HxBDD and 1,2,3,7,8,9-HxBDD [29,30]. Interestingly, in the eggs of the cormorant, only non-2,3,7,8- substituted PBDD/Fs congeners were detected [31]. In food, PBDFs occur more often than PBDDs contrarily to the congener pattern of PCDD/Fs, where dioxins occur more often than furans [27]. Brominated dioxins have also been found in the feed additive choline chloride [32]. In this case, the feed additive was screened with the DR CALUX and resulted in a suspect result without the presence of chlorinated dioxins. Investigation at that time resulted in the discovery of tetra and penta brominated dioxins and furans, with 2,3,7,8-TBDF being the most prominent congener present. The levels of 2,3,7,8-TBDF (up to 2.3 pg/g) in choline chloride were to low, since less than 1% choline chloride was added to an animal feed, it was concluded that this low concentration would not result in detectable levels in poultry eggs or meat.

Overall, PBDD/Fs are sporadically monitored compared to PCDD/Fs. Hence, a limited number of data are available for this group of compounds, and therefore human exposure is underestimated [27].

In the present study, egg and poultry meat were investigated in more detail as suspect DR CALUX outcomes were obtained. Additionally, broiler feed, feed additives, bedding material, and seaweed were investigated as possible sources of the bioactivity. The feed additives choline chloride and L-lysine are known to be used extensively in poultry feed [33,34]. Lysine is a limiting amino acid for poultry [35]. It is naturally present in soybean but is added as feed additive for economic reasons. The feed additive form mostly used is L-lysine HCl produced by fermentation by *Corynebacterium glutamicum*. Choline chloride is also being added to poultry feed as a supplement in order to increase egg production and broiler fattening [36]. In addition, seaweed was investigated as seaweed is another protein source in animal feed. Finally, the bedding material was investigated, since it has been shown to be a source of PBDD/F contamination [37] This particular bedding material is a specific product used for poultry housing in order to circumvent growth of *Salmonella* and it was shown before to be contaminated with all kinds of dioxin like AhR-agonists (results not shown). Overall, it is of utmost importance to survey and monitor all dioxin-like compounds more intensively in food and feed and additional screening with an AhR bioassay.

## 2. Materials and Methods

### 2.1. Samples

The poultry eggs, broiler meat, L-lysine (seven samples), choline chloride (two samples), broiler feed (one sample), bedding material (one sample), and seaweed (one sample) were sampled by the Netherlands Food and Consumer Product Safety Authority between 2019 and 2021 within national monitoring plans. Eggs were collected from free-range and deep litter farming.

### 2.2. Chemicals

Analytical standards and other chemicals used for the chlorinated dioxin analysis by GC-HRMS were as described previously by Ten Dam et al. [38]. For the brominated dioxins, the analytical standards as well as the ^13^C-labeled PBDD/Fs and PCDD/Fs were obtained from Cerilliant (Round Rock, TX, USA) via LGC standards. These were as follows: 1,3,7-TriBDF—2,3,7,8-TBDF—1,2,3,7,8-PeBDF—2,3,4,7,8-PeBDF—1,2,3,4,7,8-HxBDF—1,2,3,4,6,7,8-HpBDF—OBDF—1,3,7-TriBDD—2,3,7,8-TBDD—1,2,3,7,8-PeBDD—1,2,3,4,7,8HxBDD—1,2,3,6,7,8-HxBDD—1,2,3,7,8,9-HxBDD—1,2,3,4,6,7,8-HpBDD and OBDD.

### 2.3. Sample Preparation

Because dioxins are lipophilic, they accumulate in fat. Therefore, fat was extracted from egg yolk and broiler meat according to Smedes [39]. In short, sodium sulfate was added to the homogenate of egg yolk, pentane was added, and finally this mixture was filtered over sodiumsulfate, and the filtrate was evaporated under vacuum. To extract fat from broiler fat, sodiumchloride, iso-propanol and cyclohexane were added. This mixture was first ground using an ultra-turrax, and subsequently centrifuged and filtered over a sodiumsulfate filter and finally the cyclohexane was evaporated. Regarding the broiler feed and seaweed, samples were first ground using an ultra-turrax and then were extracted using an accelerated solvent extractor (ASE) (Dionex™ ASE™ 350) with hexane/acetone (1:1).

### 2.4. Sample Cleanup and Routine DR CALUX Screening

To fat aliquots of 2 g, 5 mL sulfuric acid was added, carefully mixed by rolling and left overnight in the fume hood. Next, samples were extracted twice with 10 mL hexane/diethyl ether (97:3 *v*/*v* %). The collected fraction (20 mL) was evaporated to about 2 mL using a vacuum evaporation system (Savant SPD 2010, Speed Vac Concentrator) with temperature set at 45 °C, ramp at 3, vacuum pressure at level 30 and a runtime of 30 min. Further extraction and cleanup of this 2 mL fraction on an acid-silica column were performed as described previously [40]. In short, moulds of cotton (treated to complete dryness at 160 °C) were pushed down to the tips of glass columns held in place with a clamp on a ring stand. Ten grams of acid silica was weighed into each glass column followed by 2 g of Na_2_SO_4_ (treated at 125 °C). Each column was conditioned with 20 mL hexane/diethyl ether (97:3 *v*/*v* %) and 50 mL vials were placed beneath the columns to collect each sample extract, i.e., the 2 mL extract was pipetted on the columns and the 50 mL vial was rinsed twice with 2 mL hexane/diethyl ether (97:3 *v*/*v* %), which were also brought on the column. Further extraction was performed by adding 20 mL hexane/diethyl ether (97:3 *v*/*v* %) and subsequently an extra 10 mL of the same eluent, making a total volume of about 36 mL. Regarding feed and feed additives, aliquots of 5 g were treated with 20 mL methanol/water (85/15 *v*/*v* %). Next, samples were extracted twice with 20 mL hexane/diethyl ether (97:3 *v*/*v* %). The collected fraction (40 mL) was evaporated to about 2 mL using the vacuum evaporation system with temperature set at 45 °C, ramp at 3, vacuum pressure at level 30, and a runtime of 45 min. Further extraction and cleanup of this 2 mL fraction on an acid-silica column were performed as described above for the fat extracts, resulting in a final total volume of about 36 mL for these samples. The 36 mL sample extracts coming from the acid-silica columns were evaporated to about 1 mL using the vacuum evaporation system with temperature set at 45 °C, ramp at 3, vacuum pressure at level 30, and a runtime of 45 min. Afterwards, the 1 mL was withdrawn from the vial, rinsed twice with 2 mL hexane/diethyl ether (97:3 *v*/*v* %), and transferred to a 6 mL borosilicate tube that already contained 20 µL DMSO (as a keeper). For the fat extracts, the solution was mixed before evaporation in the vacuum system with the same program but reduced runtime and 1 mL of culture medium (AMEM supplemented with 10% FBS and 0.5% penicillin/streptomycin) was added, resulting in a final DMSO concentration of approximately 2%. For the extracts prepared from feed and feed additives, the solution was mixed before evaporation in the vacuum system with the same program but reduced runtime, and an extra 20 µL of DMSO was added before 2 mL of culture medium was added, resulting in a final DMSO concentration of approximately 2%.

The DR CALUX bioassay was performed as described previously by Hoogenboom et al. [8]. In short, recombinant rat hepatoma cells (H4IIE-luc) were grown at 37 °C (5% CO_2_) and 100% relative humidity in culture medium. For analysis of the fat samples, 100 µL portions of a cell suspension (about 40,000 cells/well) were seeded in 96-well plates (Corning, New York, NY, USA) and grown for 24 h before adding 100 µL of each extract in culture medium in triplicate (final volume 200 µL, final concentration DMSO approximately 1%). For analysis of the feed and feed additive samples, 250 µL portions of a cell suspension (about 40,000 cells/well) were seeded in 48-well plates (Corning) and grown for 24 h before adding 250 µL of each extract in culture medium in triplicate (final volume 500 µL, final concentration DMSO approximately 1%). TCDD standards diluted in culture medium (final concentration DMSO 1%) were included as positive controls as well as reference butter fat samples (containing 0.59–1.01–2.07–3.07–6.19–10.20–17.90 and 35.80 pg TEQ/g fat) and reference poultry feed samples (containing 0.02–0.29–0.48–0.70–1.57 and 3.35 pg TEQ/g product).

The luciferase concentration was subsequently measured 24 h after exposure. For this, the medium was removed, and the cell monolayers were washed with 200 µL phosphate-buffered saline (PBS) (Oxoid, Basingstoke, UK). Cells were lysed using 20 µL cell culture lysis reagent (Promega, Madison, WI, USA) and incubated for 25 min at room temperature. Luciferase activity of the cell lysate was measured with a CLARIOstar microplate reader (BMG Labtech, Ortenberg, Germany) from Isogen Life Science BV (Utrecht, The Netherlands), which automatically added 100 µL assay mixture (substrate) containing 20 mM tricine, 1.07 mM (MgCO_3_)4 Mg (OH)2.5H_2_O, 2.67 mM MgSO_4_.7H_2_O, 0.1 mM EDTA, 33.3 mM DTT, 261 µM Coenzyme A, 470 µM luciferin, and 530 µM ATP at a pH of 7.8.

Dose–response curves obtained with TCDD were fitted using a user-defined exponential equation y = a0/(1 + (x/a1)^a2^) with SlideWrite Plus v.6.1 (Advanced graphics software, Pasadena, CA, USA). Where a0 is the maximum response, a1 is the concentration showing a half-maximal response (EC50) and a2 is the coefficient for the steepness of the curve. Graphs were plotted with GraphPad Prism 5. Dose–response curves obtained with the refence fat samples and the refence feed samples were fitted using y = a0 + a1 × exp(−x/a2). Using the fitted dose–reponse curves obtained with the refence fat samples and the refence feed samples, the BEQ levels in the samples were calculated.

### 2.5. Sample Cleanup for GC-HRMS and for Additional Screening with the DR CALUX Bioassay

In order to have enough sample material for multiple analyses, two pool samples were prepared for both poultry egg and broiler fat. One pool contained the fat of samples that had given a non-suspicious DR CALUX result (<1 pg BEQ/g fat) and the other pool contained the fat of samples that had given a high response in the DR CALUX (>6.2 pg BEQ/g fat) and contained no elevated levels of PCDD/F or dl-PCBs according to GC-HRMS analysis. Both “blank” pools and both “contaminated” pools were tested again in the DR CALUX, confirming that the “blank” pools contained no elevated levels of AhR-agonists (<1 pg BEQ/g fat) and that the “contaminated” pools contained levels of AhR-agonists exceeding 10 pg BEQ/g (close to the predicted BEQ level calculated from the individual samples). For GC-HRMS analysis, bedding material, broiler feed, seaweed, and the feed additives L-lysine and choline chloride were extracted using an accelerated solvent extractor (ASE) (Dionex™ ASE™ 350) with hexane/acetone (1:1).

**Figure 2:**
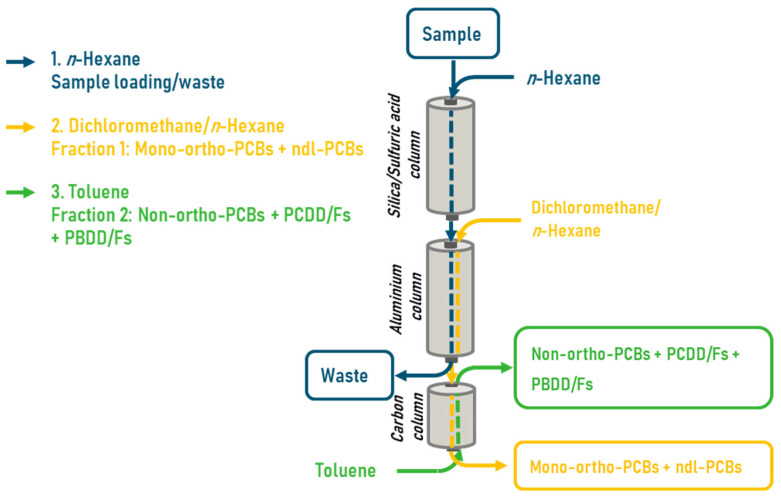
A schematic overview of the cleanup of a fat sample to extract the mono-ortho and ndl-PCBs (fraction 1) and the PCDD/Fs, PBBD/Fs, no-PCBs, and PXDD/Fs (fraction 2) using an automated DEXTech Plus system.

From the four pool samples, duplicates were prepared, one for the DR CALUX and one for the GC-HRMS analysis. For GC-HRMS analyses, 13C-labeled PBDD/Fs and PCDD/Fs were added to the fat, but not to the ones for DR CALUX analysis. For cleanup of the fat, a DEXTech Plus automated system (LCTech, Obertaufkirchen, Germany) was used (Figure 2). Therefore, 1 g fat was dissolved in 30 mL hexane and applied onto the DEXTech equipped with three columns, i.e., an acidified silica, an alumina, and a carbon column. After loading the sample on the silica column, this column and the alumina column were washed with hexane. Next, the alumina and carbon columns were eluted with 60 mL hexane/dichloromethane (1:1, *v*/*v*), which allowed the elution of mono-ortho-PCBs and ndl-PCBs, fraction 1. Then the PCDD/Fs, PBDDs/Fs, PXDD/Fs, and dl-PCB’s were eluted via a backflush with 25 mL toluene (1:1, *v*/*v*) fraction 2. The volume of the final extract was reduced to 0.5 mL using an automated evaporation system. Fraction 2 was then further evaporated to a volume of 50 μL.

The elution times for each step used was longer than with the conventional cleanup used for PCDD/Fs, respectively, 25 min instead of 10 min.

The obtained extract fractions without labeled internal standard were analyzed with the DR CALUX bioassay and the fractions with 13C-labeled internal standard with the GC-HRMS.

### 2.6. GC-HRMS Analysis

Analysis of PCDDs/Fs, PBDDs/Fs, and dioxin-like PCBs was performed as described previously by Ten Dam et al. [38]. However, the PBDDs/Fs were analyzed in a separate run and analyzed with a different GC-column. In short, extracts including the 13C-labeled compounds were analyzed on a Waters autospec high resolution mass spectrometer (Manchester, UK) coupled to an Agilent 6890 gas chromatograph (Santa Clara, SC, USA), a combi PAL autosampler from CTC (Zwingen, Switzerland), and a CIS-4 programmed temperature vaporization injector (PTV) from Gerstel (Mülheim an der Ruhr, Germany). CO_2_ cryogenic cooling was used, and the mass spectrometer was operated in electron impact ionization mode, using selected-ion monitoring (SIM) at a resolution of R = 10,000 (at 10% valley). A large volume injector (LVI) was used to inject 100 μL of the extract containing PCDD/Fs and non-ortho-PCBs on the GC while 2 μL of the extract was injected in splitless mode for the mono-ortho-PCBs and ndl-PCBs measurements for the substance groups were carried out on different GC columns. Dioxin-like PCBs and PCDD/Fs were measured on a DB5 MS (60 m × 0.25 mm × 0.25 μm) and the PBDD/Fs on a DB5 MS (5% phenyl methylpolysiloxane, 15 m × 0.2 mm × 0.1μm). All extracts were measured in SIM mode using quantifier and at least one qualifier ion for the determination of native and labeled congeners. Table 1 and Table 2 show qualifiers and quantifiers that are used for the PBDD/Fs and the PXDD/Fs measurements.

The results were corrected for recovery using the ^13^C-labeled internal standards, and the performance was evaluated through two in-house reference samples of butter fat spiked at 0.5 and 1 pg TEQ/g fat PCDD/Fs + PCBs.

### 2.7. Determination of the Relative Potency (REP) of 2,3,7,8-TBDF in the DR CALUX Bioassay

To establish the relative potency of 2,3,7,8-TBDF in the DR CALUX bioassay, a standard of 2,3,7,8-TBDF was prepared in DMSO and the concentration was checked with GC-HRMS. Then, serial dilutions of this standard were made in DMSO and each standard concentration was tested in threefold in the DR CALUX bioassay. The dose response of 2,3,7,8-TBDF was compared to that of TCDD, and after fitting, the EC_50_ value obtained for 2,3,7,8-TBDF was compared to that of TCDD and used to calculate a TEF for 2,3,7,8-TBDF.

### 2.8. Feed–Food Converter

To estimate the transfer of PBDD/Fs from feed to egg, a transfer model was used, which was developed by the National Institute for Public Health and the Environment (RIVM, Bilthoven, The Netherlands) and Wageningen Food Safety Research (WFSR, Wageningen, The Netherlands) [41].

The kinetics of the brominated dioxins might differ from the chlorinated analogues. Therefore, it is not certain if the uptake of the brominated dioxin is similar to that of its chlorinated analogue TCDD. Thus, when using the transfer kinetics of TCDD for 2,3,7,8-TBDF, the kinetic model will give an indication of the amounts of 2,3,7,8-TBDF to be found in the egg. The feed–food converter transfer model was not trained on data for broilers, and therefore it could not be used to estimate the transfer of 2,3,7,8-TBDF from feed to broiler.

## 3. Results and Discussion

### 3.1. Screening and Analysis of PCDD/Fs in Egg and Broiler

**Figure 3:**
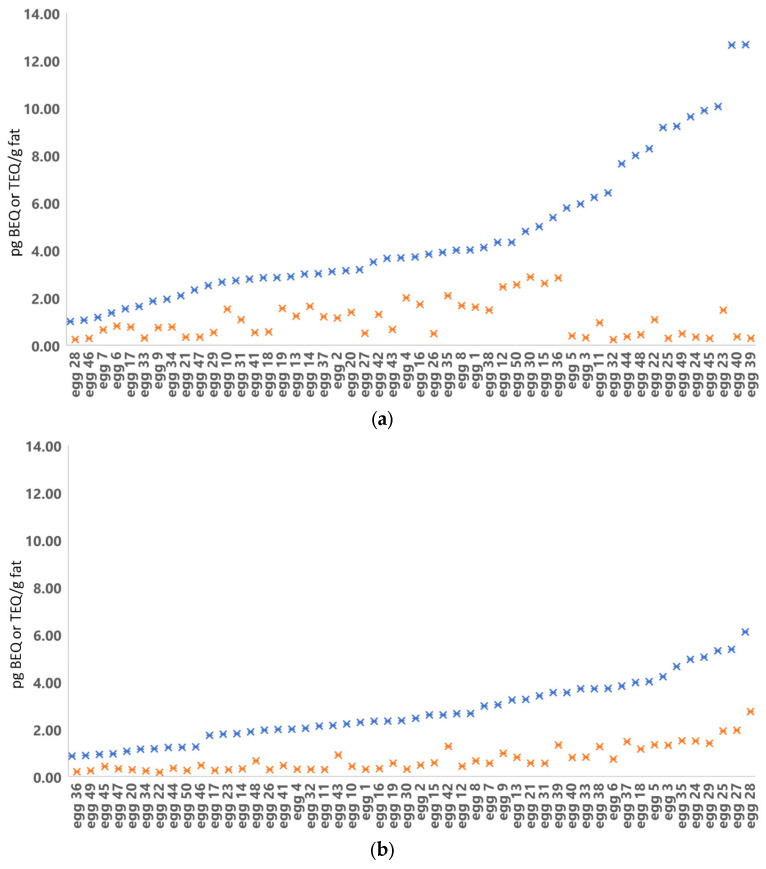
Egg sample 2019 (**a**) and 2020 (**b**) results from DR CALUX in pg BEQ/g fat (blue cross) and GC-HRMS in pg WHO (2005)-PCDD/F-PCB-TEQ/g fat (orange cross) analyses. The graph is arranged in increasing DR CALUX results.

**Figure 4:**
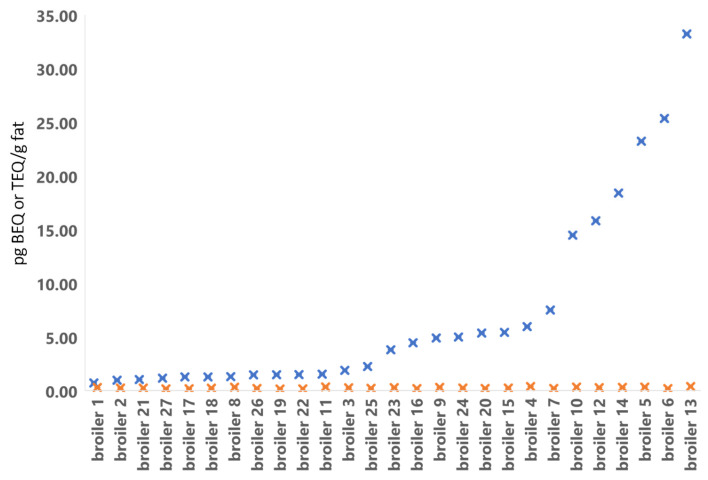
Results from DR CALUX (blue cross) in pg BEQ/g fat and GC/HRMS (orange cross) in pg WHO (2005)-PCDD/F-PCB-TEQ/g fat analyses of broiler fat from 2019.

Sample analysis using the DR CALUX bioassay for the presence of stable AhR-agonists like PCDD/Fs and dl-PCBs classifies samples as either negative (compliant) or as suspect. A sample is classified as suspect in the DR CALUX bioassay when the calculated BEQ level is above 1.75 or 1.00 pg BEQ/g fat for egg and broiler respectively. During the yearly monitoring of “primary agricultural products” in 2019, an increase in the number of both egg and broiler fat samples classified as suspect was observed compared to previous years. To illustrate, in 2017 22% of the eggs and 2% of the broiler samples were classified as suspect in the DR CALUX, and this increased in 2019 to 88% and 45% and decreased to normal levels in 2020, i.e., 36% and 4%, respectively. The increase in suspect samples in 2019 did not match the number of non-compliant samples found in confirmatory GC-HRMS analysis, i.e., the suspect screened samples indicating high levels of bioactivity did not contain elevated levels of PCDD/Fs and dl-PCBs. Figure 3a shows a clear discrepancy between the DR CALUX BEQ concentration and the GC-HRMS determined TEQ concentration of the egg samples of 2019. Meanwhile, the data from 2020 show a correlation between the DR CALUX and the GC-HRMS determined amounts (Figure 3b). In general, the DR CALUX result (BEQ content) is consistently higher than the sum TEQ content calculated from GC-HRMS analysis (TEQ content); this is due to the presence of congeners that evoke a dioxin-like response in the bioassay and thus contribute to the total BEQ response, but which are not taken into account with GC-HRMS analysis, as these congeners have no assigned TEF values. However, the data clearly elicits that several 2019 egg samples contained substantial amounts of persistent AhR-agonists that could not be explained by elevated PCDD/Fs and dl-PCBs levels (Figure 3a) or extremely high levels of congeners that have no assigned TEF values. Similarly, several 2019 broiler fat samples were shown to contain substantial amounts of persistent AhR-agonists that could not be explained by elevated PCDD/Fs and dl-PCBs levels (Figure 4). Further investigation was needed to identify the compound(s) causing the high responses in the DR CALUX bioassay.

### 3.2. Screening and Analysis of PCDD/Fs in Egg and Broiler Pool Samples

Two pools of both egg and broiler fat were prepared, i.e., pools of fat samples that were screened as negative in the DR CALUX bioassay and pools of fat that had resulted in suspect screening outcomes. The sample cleanup and extraction of the samples were similar for DR CALUX and GC-HRMS analysis to make sure that differences between the routinely used extraction or cleanup for the DR CALUX and GC-HRMS had caused the high DR CALUX response. As expected, the extracts gave either a high or low response in the DR CALUX bioassay, according to if it was a pool sample prepared from samples previously screened as negative in the bioassay or a pool sample prepared from samples previously screened as suspect in the bioassay (Table 3).

### 3.3. GC-HRMS Analysis of PBDD/Fs and Mixed Chlorinated-Brominated PXDD/Fs

**Figure 5:**
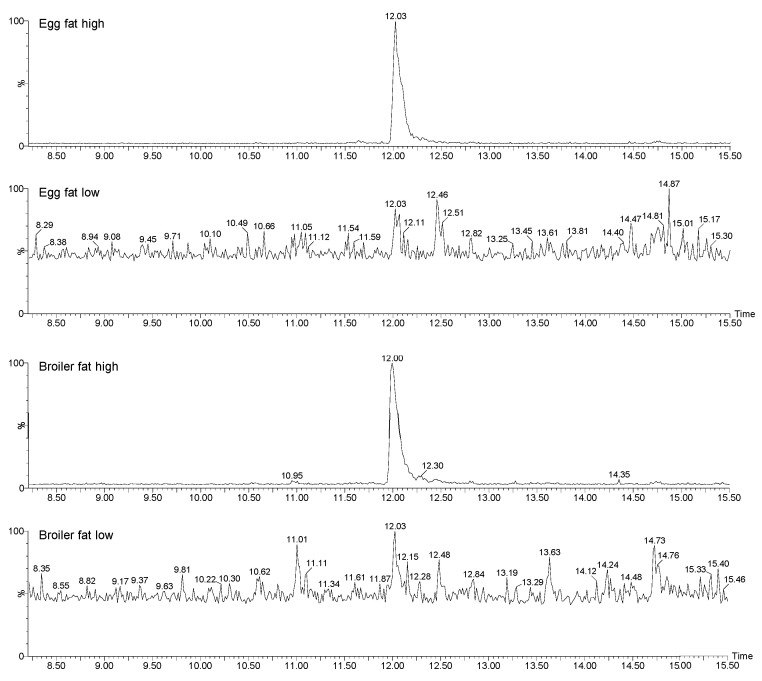
The GC-HRMS chromatograms of 2,3,7,8-TBDF in broiler and egg fat pool samples that had a high and low response in the DR CALUX.

In order to investigate if brominated dioxins could be responsible for the high DR CALUX responses in egg and broiler fat, the four pool samples were subsequently analyzed by GC-HRMS focusing on the presence of PBDDs/Fs and mixed chlorinated and brominated dioxins. From the results, it became clear that the egg and broiler pool samples that were prepared from samples having high responses in the DR CALUX, i.e., pools containing 10.45 and 18.58 pg BEQ/g fat, respectively (Table 3), both contained substantial amounts of 2,3,7,8-tetrabromodibenzofuran (2,3,7,8-TBDF), i.e., 26 and 32 pg/g fat (Figure 5). The pool samples that were prepared from samples eliciting a low response in the DR CALUX, i.e., 0.55 and 0.02 pg BEQ/g fat for egg and broiler (Table 3), did not contain 2,3,7,8-TBDF (Figure 5) above the Limit of Detection (LOD). Besides 2,3,7,8-TBDF, no other PBDDs/Fs congeners (Table 1) were detected in the egg and broiler fat.

### 3.4. Determination of the REP of 2,3,7,8-TBDF in the DR CALUX Bioassay

**Figure 6:**
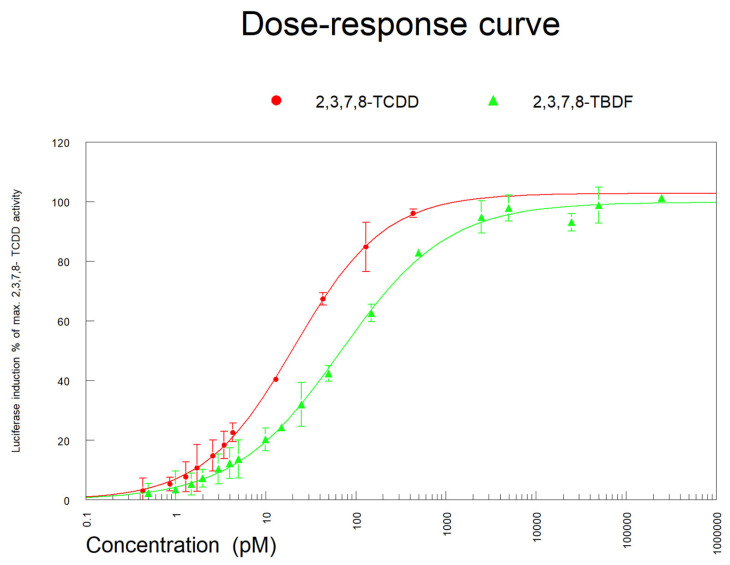
The dose responses of 2,3,7,8-TCDD and 2,3,7,8-TBDF as obtained in the DR CALUX bioassay.

The dose responses of 2,3,7,8-TCDD and 2,3,7,8-TBDF were obtained with the DR CALUX bioassay. After fitting, EC_50_ values of 20.67 and 68.12 pM were calculated for TCDD and TBDF, respectively, resulting in a REP of 0.30 for 2,3,7,8-TBDF (based on calculated EC_20_ values, a REP of 0.40 was determined for 2,3,7,8-TBDF) (Figure 6). Previously a higher REP of 0.98 was determined in the DR CALUX for 2,3,7,8-TBDF, while TBDF showed a REP of 0.86 in a DR CALUX-like assay based on a human liver cell line [15]. The REP as determined in the DR CALUX bioassay in the present study was checked by full scan HRMS measurement of the 2,3,7,8-TBDF standard and confirmed the concentrations as used to determine the dose responses depicted in Figure 6.

### 3.5. Eliciting the Source of the 2,3,7,8-TBDF Contamination

To determine the possible source of the 2,3,7,8-TBDF contaminated eggs and broilers, we analyzed L-lysine and cholinde chloride (feed additives), poultry feed, bedding material, and seaweed samples from 2019, which also showed elevated responses in the DR CALUX bioassay. In addition, two recent choline chloride samples (from 2021) that showed an elevated DR CALUX response were further analyzed (no suspect screened choline chloride samples from 2019 or 2020 were available).

The bedding material and seaweed sample did not contain 2,3,7,8-TBDF. The seaweed did contain tribromo-dibenzofurans but also tetrabromo-dibenzofurans although not the 2,3,7,8-substituted congener. The bedding material contained none of the PBDD/Fs, but contained all the regulated PCDD/Fs, mixed PXDD/Fs, and also tribromo-dibenzofurans and tribromo-dibenzo-p-dioxins. Just as the seaweed, this bedding material is unlikely the source of the present 2,3,7,8-TBDF contamination of eggs and broilers, but it is heavily contaminated with regulated dioxins and mixed PXDD/Fs and is prohibited for use in the European Union. On the other hand, the poultry feed and the two feed additives choline chloride and L-lysine contained 2,3,7,8-TBDF. Especially the poultry feed sample and several L-lysine samples contained high amounts of 2,3,7,8-TBDF. Just like the contaminated egg and broiler fat samples, the poultry feed only contained TBDF, whereas the feed additives also contained other PBDF congeners, although always at lower levels than TBDF, i.e., 1,2,3,7,8-PeBDF in both choline chloride and L-lysine and in addition 2,3,4,7,8-PeBDF, 1,2,3,4,7,8-HxBDF and 1,2,3,4,6,7,8-HpBDF in several L-lysine samples (Table 4). Only two samples (both lysine) contained a PBDD, i.e., 2,3,7,8,-TBDD and 1,2,3,7,8,9-HxBDD, but both at low levels. Overall, this strongly indicates that the poultry feed was the source of the contaminated eggs and broilers.

The high broiler fat pool contained 31 pg 2,3,7,8,-TBDF/g fat according to GC-HRMS analysis (Table 4). As the DR CALUX 2,3,7,8-TBDF displays a REP of about 0.4, the by GC-HRMS determined amount would theoretically result in 12.4 pg BEQ/g fat, which is in line with the determined amount of 18.6 pg BEQ/g fat in the DR CALUX (Table 3). The same holds true for the high egg pool sample, which contained 21 pg 2,3,7,8-TBDF/g fat according to GC-HRMS analysis (Table 4), which would theoretically result in 8.4 pg BEQ/g fat, which is in line with the determined amount of 10.5 pg BEQ/g fat in the DR CALUX (Table 3). As stated before, in general the DR CALUX result (BEQ content) is consistently higher than the sum TEQ content calculated from GC-HRMS analysis (TEQ content), due to the presence of congeners that evoke a dioxin-like response in the bioassay and thus contribute to the total BEQ response, but which are not taken into account with GC-HRMS analysis, as these congeners have no assigned TEF values or by congeners that are overseen by GC-HRMS analysis, like in the case of the present contaminations with 2,3,7,8-TBDF. Besides other halogenated PXDD/Fs and dl-PXBs, this might include halogenated PAHs [42] or mixed dioxins. Mixed dioxins such as a dichlorodibromodibenzo-p-dioxin and a monobromo-tetrachlorodibenzofuran were detected in egg and broiler, although in very low concentrations. The positions of the bromine and chlorine on the molecule are unknown, due to lack of congener specific standards.

### 3.6. Feed-to-Food Converter

The presence of 2,3,7,8,-TBDF in both “egg pool high”, “broiler pool high” and the “poultry feed” indicates that the poultry feed was the potential source of the contaminated eggs and broilers. The actual amount of feed additives added to the poultry feed was not known. Assuming this to be 1%, the amount of 2,3,7,8,-TBDF found in poultry feed (3.6 pg/g) cannot be originated from these particular additives unless the amount of L-lysine in the feed was above 10%, unless the feed additives that were added to the poultry feed contained higher amounts of TBDF than what was found in the feed additives from this study.

This further proves that this contaminated poultry feed could indeed be the source of the contaminated eggs and poultry meat, which was obtained by predicting the amounts of 2,3,7,8-TBDF in egg fat when poultry is fed with this contaminated feed containing 3.6 pg TBDF/g product. Using the RIVM/WFSR feed–food converter model for chlorinated dioxins and furans [41], the amount of 2,3,7,8-TBDF that would end up in egg was calculated. As the kinetics of 2,3,7,8-TBDF are not known, we assumed the same kinetics for 2,3,7,8-TBDF as for its chlorinated analogue and a feed intake of 0.065 kg/day. The feed–food converter predicted a maximum of 32 pg TBDF/g fat in egg yolk fat, which corresponds with the content as determined in the “egg pool high” samples, i.e., 21 pg TBDF/g fat.

## 4. Conclusions

The present study shows that the DR CALUX indicated several samples as suspect, while they were compliant according to GC-HRMS analysis for PCDD/F and dl-PCBs. Upon further investigation, it turned out that egg and broiler were heavily contaminated with 2,3,7,8-TBDF and that the source was probably a contaminated poultry feed. From this, it can be concluded that by including bioassays in sample analysis offers several advantages over using targeted MS methods only. In general, effect-based bioassays will detect both known and unknown bioactive compounds. The DR CALUX bioassay detects all active AhR-agonists present in a sample extract. This includes the known PCDD/Fs and dl-PCBs, but also the PBDD/Fs, dl-PBBs, PAHs, halogenated PAHs, and PCNs [42]. Bioassays like the DR CALUX thus have the potential to detect new and emerging risks.

In addition, all feed additives contained PBDDs/Fs, which is rather alarming and more research is needed on how these brominated dioxins can end up in these feed additives. Both PBDD/Fs as well as PXDD/Fs are poorly monitored in agricultural products throughout Europe. Only in some extreme cases and often only by laboratories using effect-based screening assays, such as in the present case, are these compounds detected. Therefore, it is advised to include the use of bioassays in monitoring programs or to include both PBDD/FS and PXDD/Fs in the applied GC-HRMS analyses.

## Figures and Tables

**Table 1:** Measured masses (*m*/*z*) of the brominated dioxins and furans including the internal standards.

Congener	Elemental Composition	Qualifier (*m*/*z*)	Quantifier (*m*/*z*)	Internal Standard	Qualifier (*m*/*z*)
1,3,7-Tribromodibenzofuran	C_12_H_5_Br_3_O	403.788	405.785	^13^C-2,3,7,8-TBDF	491.723
2,3,7,8-TBDF	C_12_H_4_Br_4_O	483.695	481.697	^13^C-2,3,7,8-TBDF	491.723
1,2,3,7,8-PeBDF	C_12_H_3_Br_5_O	561.606	563.604	^13^C-1,2,3,7,8-PeBDF	569.633
2,3,4,7,8-PeBDF	C_12_H_3_Br_5_O	561.606	563.604	^13^C-2,3,4,7,8-PeBDF	569.633
1,2,3,4,7,8-HxBDF	C_12_H_2_Br_6_O	641.514	643.512	^13^C-1,2,3,4,7,8-HxBDF	649.542
1,2,3,4,6,7,8-HpBDF	C_12_H_1_Br_7_O	719.425	721.423	^13^C-1,2,3,4,6,7,8-HpBDF	727.452
OBDF	C_12_Br_8_O	799.333	797.335	^13^C-1,2,3,4,6,7,8-HpBDF	727.452
1,3,7-Tribromodibenzo-p-dioxin	C_12_H_5_Br_3_O_2_	419.782	421.780	^13^C-2,3,7,8-TBDD	507.718
2,3,7,8-TBDD	C_12_H_4_Br_4_O_2_	499.690	497.692	^13^C-2,3,7,8-TBDD	507.718
1,2,3,7,8-PeBDD	C_12_H_3_Br_5_O_2_	577.600	579.598	^13^C-1,2,3,7,8-PeBDD	585.628
1,2,3,4,7,8-1,2,3,6,7,8-HxBDD	C_12_H_2_Br_6_O_2_	657.509	655.511	^13^C-1,2,3,7,8,9-HxBDD	665.537
1,2,3,7,8,9-HxBDD	C_12_H_2_Br_6_O_2_	657.509	655.511	^13^C-1,2,3,4,7,8-HxBDD	665.537
1,2,3,4,6,7,8-HpBDD	C_12_H_1_Br_7_O_2_	735.419	737.417	^13^C-1,2,3,4,6,7,8-HpBDD	743.447
OBDD	C_12_Br_8_O_2_	815.328	813.330	^13^C-OBDD	823.356

**Table 2:** Measured masses (*m*/*z*) of the mixed dioxins and furans including the internal standards.

Congener	Elemental Composition	Qualifier (*m*/*z*)	Quantifier (*m*/*z*)	Internal Standard	Qualifier (*m*/*z*)
2,8-dibromo-3,7-dichloro-dibenzo-p-dioxin	C_12_H_4_Br_2_Cl_2_O_2_	409.7933	411.791	^13^C-2,3,7,8-TBDF	491.723
8-bromo-2,3,4-trchlorodibenzofuran	C_12_H_4_BrCl_3_O	349.8487	351.846	^13^C-2,3,7,8-TBDF	491.723
4-bromo-2,4,7,8-tetrachlorodibenzofuran	C_12_H_3_BrCl_4_O	383.8096	385.8069	^13^C-1,2,3,7,8-PeBDF	569.633
2-bromo-3,7,8-trichlorodibenzofuran	C_12_H_4_BrCl_3_O	349.8487	351.846	^13^C-2,3,4,7,8-PeBDF	569.633
2-bromo-1,3,7,8,-tetrachlorodibenzofuran	C_12_H_3_BrCl_4_O	383.8096	385.8069	^13^C-1,2,3,4,7,8-HxBDF	649.542
2,3-dibromo-7,8-dichlorodibenzo-p-dioxin	C_12_H_4_Br_2_Cl_2_O_2_	409.7933	411.791	^13^C-1,2,3,4,6,7,8-HpBDF	727.452
2-bromo-1,3,7,8,-tetrachlorodibenzo-p-dioxin	C₁₂H₃BrCl₄O₂	399.8045	401.8019	^13^C-1,2,3,4,6,7,8-HpBDF	727.452

**Table 3:** DR CALUX results from four pool samples i.e., eggs low, eggs high, and broiler low, broiler high.

Sample	DR CALUX Result	Assessment
Eggs low	0.55 pg BEQ/g	Negative
Eggs high	10.45 pg BEQ/g	Suspect
Broiler low	0.02 pg BEQ/g	Negative
Broiler high	18.58 pg BEQ/g	Suspect

**Table 4:** Results from DR CALUX and GC/HRMS analyses of pool samples, broiler- and egg fat-, choline chloride, poultry feed, and L-lysine. Expressed in bioanalytical equivalent (BEQ), WHO 2005 sum PCDD/F-PCB-TEQ and PBDD/F congener per pg/g fat or product.

Sample Type	DR CALUX ng BEQ/kg	GC/HRMS PCDD/Fs + PCBs ng TEQ/kg	GC/HRMS PBDD/F pg/g Fat/Product						
			2,3,7,8 TBDF	1,2,3,7,8 PeBDF	2,3,4,7,8 PeBDF	1,2,3,4,7,8HxBDF	1,2,3,4,6,7,8HpBDF	2,3,7,8 TBDD	1,2,3,7,8,9HxBDD
Broiler fat (pool) high	19	0.03	31	^1^	^1^	^1^	^1^	^1^	^1^
Broiler fat (pool) low	0.02								
Egg fat (pool) high	10	0.16	21	^1^	^1^	^1^	^1^	^1^	^1^
Egg fat (pool) low	0.55								
Choline chloride	1	0.045	0.23	^1^	^1^	^1^	^1^	^1^	^1^
Choline chloride	3	0.044	3.13	0.41	^1^	^1^	^1^	^1^	^1^
Poultry feed	4	0.103	3.6	^1^	^1^	^1^	^1^	^1^	^1^
L-lysine	7	0.039	1.86	1.59	^1^	1.04	^1^	^1^	^1^
L-lysine	15	0.449	2.39	1.71	0.26	0.25	^1^	^1^	^1^
L-lysine	16	0.155	4.92	1.47	0.26	2	0.21	^1^	0.08
L-lysine	19	0.062	5.34	2.09	0.31	^1^	^1^	^1^	^1^
L-lysine	22	0.248	8.33	2.53	0.27	1.11	0.15	^1^	^1^
L-lysine	22	0.508	8.8	3.53	0.62	0.61	^1^	^1^	^1^
L-lysine	45	0.243	22.3	0.13	^1^	^1^	^1^	0.05	^1^
Bedding material	750	143	^1^	^1^	^1^	^1^	^1^	^1^	^1^
Seaweed (me hijiki)	2.24	0.15	^1^	^1^	^1^	^1^	^1^	^1^	^1^
Butter fat 0.5	0.59	0.36	^1^	^1^	^1^	^1^	^1^	^1^	^1^
Butter fat 6.0	6.19	4.5	^1^	^1^	^1^	^1^	^1^	^1^	^1^

^1^ <limit of quantification.

## Data Availability

The original contributions presented in the study are included in the article, further inquiries can be directed to the corresponding author.

## References

[1] Kan C.A., Meijer G.A.L. (2007). The risk of contamination of food with toxic substances present in animal feed. Anim. Feed. Sci. Technol..

[2] Tóth G., Hermann T., Da Silva M., Montanarella L. (2016). Heavy metals in agricultural soils of the European Union with implications for food safety. Environ. Int..

[3] Oplatowska-Stachowiak M., Elliott C.T. (2017). Food colors: Existing and emerging food safety concerns. Crit. Rev. Food Sci. Nutr..

[4] Gerssen A., Bovee T.H., van Ginkel L.A., van Iersel M.L., Hoogenboom R.L. (2019). Food and feed safety: Cases and approaches to identify the responsible toxins and toxicants. Food Control.

[5] Hites R.A. (2011). Dioxins: An Overview and History. Environ. Sci. Technol..

[6] Horii Y., van Bavel B., Kannan K., Petrick G., Nachtigall K., Yamashita N. (2008). Novel evidence for natural formation of dioxins in ball clay. Chemosphere.

[7] Van Den Berg M., Birnbaum L.S., Denison M., De Vito M., Farland W., Feeley M., Fiedler H., Håkansson H., Hanberg A., Haws L. (2006). The 2005 World Health Organization reevaluation of human and Mammalian toxic equivalency factors for dioxins and dioxin-like compounds. Toxicol. Sci..

[8] Hoogenboom L., Traag W., Bovee T., Goeyens L., Carbonnelle S., Vanloco J., Beernaert H., Jacobs G., Schoeters G., van Loco J. (2006). The CALUX bioassay: Current status of its application to screening food and feed. TrAC Trends Anal. Chem..

[9] Larigot L., Juricek L., Dairou J., Coumoul X. (2018). AhR signaling pathways and regulatory functions. Biochim. Open.

[10] Malisch R., Kotz A. (2014). Dioxins and PCBs in feed and food—Review from European perspective. Sci. Total Environ..

[11] Srogi K. (2008). Levels and congener distributions of PCDDs, PCDFs and dioxin-like PCBs in environmental and human samples: A review. Environ. Chem. Lett..

[12] Ssebugere P., Sillanpää M., Matovu H., Mubiru E. (2019). Human and environmental exposure to PCDD/Fs and dioxin-like PCBs in Africa: A review. Chemosphere.

[13] Behnisch P.A., Hosoe K., Sakai S. (2003). Brominated dioxin-like compounds: In vitro assessment in comparison to classical dioxin-like compounds and other polyaromatic compounds. Environ. Int..

[14] van den Berg M., Denison M.S., Birnbaum L.S., Devito M.J., Fiedler H., Falandysz J., Rose M., Schrenk D., Safe S., Tohyama C. (2013). Polybrominated dibenzo-p-dioxins, dibenzofurans, and biphenyls: Inclusion in the toxicity equivalency factor concept for dioxin-like compounds. Toxicol. Sci..

[15] Budin C., Petrlik J., Strakova J., Hamm S., Beeler B., Behnisch P., Besselink H., van der Burg B., Brouwer A. (2020). Detection of high PBDD/Fs levels and dioxin-like activity in toys using a combination of GC-HRMS, rat-based and human-based DR CALUX^®^ reporter gene assays. Chemosphere.

[16] Birnbaum L.S., Staskal D.F., Diliberto J.J. (2003). Health effects of polybrominated dibenzo-p-dioxins (PBDDs) and dibenzofurans (PBDFs). Environ. Int..

[17] Fromme H., Hilger B., Albrecht M., Gries W., Leng G., Völkel W. (2016). Occurrence of chlorinated and brominated dioxins/furans, PCBs, and brominated flame retardants in blood of German adults. Int. J. Hyg. Environ. Health.

[18] Yang J., Yu H., Xie Z., Yang Y., Zheng X., Zhang J., Huang Q., Wen T., Wang J. (2020). Pathways and influential factors study on the formation of PBDD/Fs during co-processing BDE-209 in cement kiln simulation system. Ecotoxicol. Environ. Saf..

[19] Hanari N., Kannan K., Miyake Y., Okazawa T., Kodavanti P.R., Aldous K.M., Yamashita N. (2006). Occurrence of polybrominated biphenyls, polybrominated dibenzo-p-dioxins, and polybrominated dibenzofurans as impurities in commercial polybrominated diphenyl ether mixtures. Environ. Sci. Technol..

[20] Schlummer M., Mäurer A., Leitner T., Spruzina W. (2006). Report: Recycling of flame-retarded plastics from waste electric and electronic equipment (WEEE). Waste Manag. Res..

[21] Fernandes A.R., Lake I.R., Dowding A., Rose M., Jones N.R., Smith F., Panton S. (2023). The transfer of environmental contaminants (Brominated and Chlorinated dioxins and biphenyls, PBDEs, HBCDDs, PCNs and PFAS) from recycled materials used for bedding to the eggs and tissues of chickens. Sci. Total Environ..

[22] Fernandes A., D’Silva K., Driffield M., White S., Rose M. (2005). Brominated Flame Retardants and Brominated Dioxins in 2003 Total Diet Samples.

[23] Fernandes A., Lake I., Dowding A., Rose M., Jones N., Petch R., Smith F., Panton S. (2019). The potential of recycled materials used in agriculture to contaminate food through uptake by livestock. Sci. Total Environ..

[24] Mwangi J.K., Lee W.-J., Wang L.-C., Sung P.-J., Fang L.-S., Lee Y.-Y., Chang-Chien G.-P. (2016). Persistent organic pollutants in the Antarctic coastal environment and their bioaccumulation in penguins. Environ. Pollut..

[25] Bjurlid F., Kärrman A., Ricklund N., Hagberg J. (2017). Occurrence of brominated dioxins in a study using various firefighting methods. Sci. Total Environ..

[26] Zacs D., Rjabova J., Bartkevics V. (2013). Occurrence of brominated persistent organic pollutants (PBDD/DFs, PXDD/DFs, and PBDEs) in Baltic wild salmon (*Salmo salar*) and correlation with PCDD/DFs and PCBs. Environ. Sci. Technol..

[27] Fernandes A.R., Falandysz J. (2021). Polybrominated dibenzo-p-dioxins and furans (PBDD/Fs): Contamination in food, humans and dietary exposure. Sci. Total Environ..

[28] World Health Organization (2016). Dioxins and Their Effects on Human Health.

[29] Pajurek M., Pietron W., Maszewski S., Mikolajczyk S., Piskorska-Pliszczynska J. (2019). Poultry eggs as a source of PCDD/Fs, PCBs, PBDEs and PBDD/Fs. Chemosphere.

[30] Fernandes A., Tlustos C., Smith F., Carr M., Petch R., Rose M. (2009). Polybrominated diphenylethers (PBDEs) and brominated dioxins (PBDD/Fs) in Irish food of animal origin. Food Addit. Contam. Part B.

[31] Watanabe K., Senthilkumar K., Masunaga S., Takasuga T., Iseki N., Morita M. (2004). Brominated Organic Contaminants in the Liver and Egg of the Common Cormorants (Phalacrocorax carbo) from Japan. Environ. Sci. Technol..

[32] Malisch R. (2017). Incidents with Dioxins and PCBs in Food and Feed-Investigative Work, Risk Management and Economic Consequences. J. Environ. Prot..

[33] Waguespack A.M., Powell S., Bidner T.D., Payne R.L., Southern L.L. (2009). Effect of incremental levels of L-lysine and determination of the limiting amino acids in low crude protein corn-soybean meal diets for broilers1. Poult. Sci..

[34] Jespersen J.C., Richert S., Dorigam J.C.D.P., Oelschlager M.L., Dilger R.N. (2021). Effects of lysine biomass supplementation on growth performance and clinical indicators in broiler chickens. Poult. Sci..

[35] Gous R., Morris T. (1985). Evaluation of a diet dilution technique for measuring the response of broiler chickens to increasing concentrations of lysine. Br. Poult. Sci..

[36] Igwe I.R., Okonkwo C.J., Uzoukwu U.G., Onyenegecha C.O. (2015). The Effect of Choline Chloride on the Performance of Broiler Chickens. Annu. Res. Rev. Biol..

[37] Hahladakis J.N., Velis C.A., Weber R., Iacovidou E., Purnell P. (2018). An overview of chemical additives present in plastics: Migration, release, fate and environmental impact during their use, disposal and recycling. J. Hazard. Mater..

[38] Dam G.T., Pussente I.C., Scholl G., Eppe G., Schaechtele A., van Leeuwen S. (2016). The performance of atmospheric pressure gas chromatography-tandem mass spectrometry compared to gas chromatography-high resolution mass spectrometry for the analysis of polychlorinated dioxins and polychlorinated biphenyls in food and feed samples. J. Chromatogr. A.

[39] Smedes F. (1999). Determination of Total Lipid Using Non-Chlorinated Solvents. Analyst.

[40] Akuamoa F., Hoogenboom R.L., Weide Y., van der Weg G., Rietjens I.M., Bovee T.F. (2022). Presence and risks of polycyclic aromatic hydrocarbons, dioxins and dioxin-like PCBs in dietary plant supplements as elucidated by a combined DR CALUX^®^ bioassay and GC-HRMS based approach. Food Addit. Contam. Part A.

[41] Notenboom S., Punt A., Hoogenveen R., Zeilmaker M.J., Hoogenboom R.L.A.P., Bokkers B.G.H. (2023). A congener-specific modelling approach for the transfer of polychlorinated dibenzo-p-dioxins and dibenzofurans and dioxin-like polychlorinated biphenyls from feed to eggs of laying hens. Food Addit. Contam. Part A.

[42] Zhou Y., van Leeuwen S.P., Knobloch M., Dirks C., Weide Y., Bovee T.F. (2021). Impurities in technical mixtures of chlorinated paraffins show AhR agonist properties as determined by the DR-CALUX bioassay. Toxicol. Vitr..

